# Risk factors for non‐gastric‐cancer‐related death after gastrectomy in elderly patients

**DOI:** 10.1002/ags3.12588

**Published:** 2022-06-20

**Authors:** Michihisa Iida, Shigeru Takeda, Chiyo Nakashima, Mitsuo Nishiyama, Yusaku Watanabe, Nobuaki Suzuki, Shigefumi Yoshino, Yuki Nakagami, Tsuyoshi Tanabe, Hiroaki Nagano

**Affiliations:** ^1^ Department of Gastroenterological, Breast and Endocrine Surgery Yamaguchi University Graduate School of Medicine Yamaguchi Japan; ^2^ National Hospital Organization Kanmon Medical Center Yamaguchi Japan; ^3^ Department of Public Health and Preventive Medicine Yamaguchi University Graduate School of Medicine Ube Japan

**Keywords:** elderly, gastrectomy, gastric cancer, prognosis, risk factors

## Abstract

**Aim:**

To identify preoperative factors, especially other diseases that cause death, that are associated with the prognosis of gastrectomy in elderly patients with gastric cancer.

**Methods:**

This retrospective study included a total of 211 consecutive patients aged ≥75 years who underwent radical gastrectomy due to gastric cancer. Time‐dependent receiver operating characteristic curve analysis was performed to determine the optimal cutoff values for various perioperative factors. Risk factors for the overall survival and death from other diseases were analyzed using the Cox proportional hazards model.

**Results:**

Among the all perioperative factors, sex, neutrophil‐to‐lymphocyte ratio, skeletal muscle mass index, and lymph node dissection in accordance with guidelines or not extracted as independent risk factors for death from other diseases. In an analysis restricted to the preoperative factors, sex, neutrophil‐to‐lymphocyte ratio, and skeletal muscle mass index of the patients were extracted as independent risk factors for death from other diseases and overall survival. We divided the patients into four groups according to the number of preoperative risk factors for death from other diseases and found that the 5‐year non‐gastric‐cancer‐related survival was different among the four groups (risk factor 0, 91.7%; risk factor 1, 83.3%; risk factor 2, 56.3%; risk factor 3, 27.2%; *P* < 0.001).

**Conclusion:**

Male sex, low skeletal muscle mass index, and high neutrophil‐to‐lymphocyte ratio are risk factors for non‐gastric‐cancer‐related death and the overall survival of elderly patients undergoing gastrectomy. Cautious treatment strategies are needed for elderly gastric cancer patients with many risk factors.

## Graphical abstract

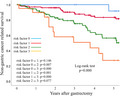

## 1 INTRODUCTION

With the aging of the world population, the number of elderly cancer patients is increasing.^1^ The risk of developing gastric cancer is higher in elderly patients than in younger patients,^2^, ^3^ and the proportion of elderly patients diagnosed with gastric cancer is increasing every year.^4^ In Japan, which has one of the most aged populations in the world,^5^ there is a growing interest in surgical strategies for elderly patients with gastric cancer. Recent reports have shown that the short‐term results of surgery for elderly gastric cancer patients are generally feasible^6^ ^,^ ^7^ but there are still insufficient reports on the long‐term prognosis of surgery in elderly gastric cancer patients. Elderly patients are often treated with non‐standard treatment, with off‐label endoscopic submucosal dissection or reduced lymphadenectomy, rather than a standard treatment based on the patient's general condition.^7^, ^8^ However, there are no clear criteria for the selection of patients to be treated in an abortive manner. Prediction of the long‐term prognosis based on preoperative factors may allow us to choose a treatment that is more appropriate for the patient's condition.

Nunobe et al reported that the ratio of deaths after gastrectomy for stage I gastric cancer in elderly patients over 75 years of age was higher due to other diseases rather than due to gastric cancer, and this ratio increased with age.^9^ This indicates the need to pay attention not only to deaths from gastric cancer but also to deaths from diseases other than gastric cancer, when considering the postoperative survival of elderly patients after gastric cancer surgery. Although many risk factors, such as tumor progression and the occurrence of postoperative complications for cancer‐related death after gastrectomy have already been reported,^10^, ^11^ the risk factors for death from other diseases after gastrectomy have been reported only in a few cases^12^, ^13^ and have not been fully elucidated. The purpose of this study was to identify preoperative risk factors for death in elderly patients after gastrectomy, especially non‐gastric cancer‐related death.

## 2 METHODS

### 2.1 Patients

This retrospective study included 217 consecutive patients over 75 years of age who were pathologically diagnosed with stage I‐III primary gastric cancer and underwent curative gastrectomy at the Yamaguchi University Medical Hospital (Yamaguchi, Japan) between January of 2007 and December of 2019. Six patients were excluded as either their preoperative computed tomography (CT) results were unavailable (three patients) or they had simultaneous double cancer (three patients). This study was approved by the institutional review board of the Yamaguchi University Hospital (H28‐182).

### 2.2 Preoperative parameters

Demographics (age, sex), co‐morbidities (modified frailty index [mFI],^14^ Charlson comorbidity index [CCI]), and performance status (PS) data were obtained from medical records. Body mass index (BMI) was calculated as body weight (kg)/height (m^2^). Preoperative laboratory data such as the complete blood count and serum albumin were routinely measured within the 2 weeks before surgery. Laboratory‐related parameters were calculated as follows: prognostic nutritional index (PNI) = serum albumin value (g/L) + 0·005 × total lymphocyte count in the peripheral blood (per mm3); neutrophil‐to‐lymphocyte ratio (NLR) = neutrophil count/lymphocyte count; platelet‐to‐lymphocyte ratio (PLR) = platelet count/lymphocyte count. Multidetector computed tomography (MDCT) was performed for all patients within 4 weeks before the surgery, and body composition parameters such as the visceral fat area (VFA) and skeletal muscle area (SMA) were measured from the MDCT image using a fat rate software (AZE Virtual Place, Aze Ltd. Tokyo, Japan), as described in our previous study.^15^ The VFA was defined as the area of fat at the level of the umbilicus, measured by preoperative MDCT. The SMA was defined as the area of the abdominal muscles, psoas, and paraspinal muscles, measured using axial slices at the level of the third lumbar vertebra. The skeletal muscle index (SMI) was calculated as SMA divided by height of the body squared. Preoperative N factor was evaluated by preoperative CT, and metastatic nodes were diagnosed as having a short axis diameter ≥ 10 mm or round node with a short diameter 5‐9 mm.

Histological type was classified into differentiated and undifferentiated, and depth of tumor invasion (T factor), lymph node metastasis (N factor), and stage were described according to the 3rd English edition of the Japanese Classification of Gastric Carcinoma. The severity of postoperative complications was determined by the Clavien‐Dindo (CD) classification, patients with grade II or higher were defined as having postoperative complications.^16^

The assessed primary and secondary outcomes were used to identify the preoperative factors associated with the non‐gastric‐cancer‐related survival (NGCaS) and overall survival (OS) after gastrectomy for elderly patients. The NGCaS was measured from surgery to death from a non‐gastric‐cancer‐related cause, and deaths due to gastric cancer were treated as censored. The OS was measured from surgery to death from any cause or the last follow‐up.

### 2.3 Surgical procedure

All patients underwent either distal gastrectomy (DG), total gastrectomy (TG), or proximal gastrectomy (PG) with D1, D1+, or D2 lymphadenectomy according to the 3rd English edition of the Japanese guidelines. In principle, lymph node dissection was performed according to these guidelines, but in some cases, surgeons used their discretion on whether or not to perform the lymph node dissection according to the guidelines depending on poor general condition. In cases of poor general condition, limited lymph node dissection was often performed. We subsequently evaluated whether the extent of lymph node dissection was standard or not. Dissection performed in accordance with the guideline recommendations on lymph node dissection was defined as standard lymph node dissection. Otherwise, lymph node dissection was defined as reduced lymph node dissection. Billroth I, Billroth II, or Roux‐en Y reconstruction were performed for DG, Roux‐en Y reconstruction was performed for TG, and double tract reconstruction was performed for PG cases.

### 2.4 Statistical analysis

Each optimal cut‐off value was used to determine the optimal cut‐off values of age, PS, mFI, CCI, PNI, NLR, PLR, BMI, VFA, SMI, operative duration, and blood loss using the time‐dependent receiver operating characteristic (ROC) curve analysis, the Kaplan‐Meier (KM) estimation method, and the closest‐to‐top left index. The NGCaS and OS were calculated using Kaplan‐Meier methods, and the survival curves were compared using the log‐rank test. Uni‐ and multivariate analyses for NGCaS and OS were conducted with Cox proportional hazards models. The hazard ratios (HRs) and 95% confidence intervals (CIs) were calculated. A *P*‐value of <0.05 was considered statistically significant. All statistical analyses were performed using SPSS version 25.0 (SPSS Inc., Chicago, IL, USA), with the exception of the time‐dependent ROC curve‐analysis, which was performed using the statistical programming language R for 64‐bit Windows (version 4.1.0, R Development Core Team).

## 3 RESULTS

### 3.1 Diagnostic accuracy and cutoffs of perioperative parameters

To determine the cut‐off values and the area under the ROC curves (AUCs) of the preoperative factors for OS and NGCaS, time‐dependent ROC analysis was performed. AUC and optimal cut‐off value for the OS of each perioperative parameter are shown in Figure S1. AUC and optimal cut‐off value for the NGCaS of each perioperative parameter are shown in Figure S2.

### 3.2 Clinicopathological findings

The mean patient age was 80.1 years (75‐94), and 64.5% of the patients were male. In the CCI, 64.5% of the patients had some comorbidity with a score of 1 or more. The pathological stages were I, II, and III in 63.5%, 18.5%, and 18% of the patients, respectively. The operation modes were distal gastrectomy, total gastrectomy, and proximal gastrectomy in 68.2%, 29.9%, and 1.9% of the patients, respectively, with laparoscopy in 67.8% and laparotomy in 32.2% of the patients. For guideline‐based lymph node dissection, standard lymph node dissection was performed in 86.7% of patients and reduced lymph node dissection in 13.3% of patients. Further details of the clinicopathological findings are presented in Table 1.

**TABLE 1:** Clinicopathological findings of patients

Characteristics	Number of patients	Percentage
Mean age (years)	80.1 ± 4.1 (75‐94)	
Gender		
Male	136	64.5
Female	75	35.5
PS		
0	159	75.4
1	41	19.4
2	10	4.7
3	1	0.5
Modified frailty index		
0	34	16.1
1	93	44.1
2	58	27.5
≥3	26	12.3
Charlson comorbidity index		
0	75	35.5
1	66	31.2
2	39	18.5
≥3	31	14.7
PNI	46.9 ± 5.7 (28.1‐60.6)	
NLR	2.9 ± 2.2 (0.6‐25.4)	
PLR	154.9 ± 82.5 (26.4‐698.3)	
BMI (kg/m^2^)	22.0 ± 3.3 (14.2‐32.8)	
VFA (cm^2^)	128.5 ± 66.5 (8.3‐395.1)	
SMI (cm^2^/m^2^)	41.7 ± 7.2 (27.8‐65.8)	
Type of resection		
Distal gastrectomy	144	68.2
Proximal gastrectomy	4	1.9
Total gastrectomy	63	29.9
Approach		
Open	68	32.2
Laparoscopy	143	67.8
Extent of node dissection		
D1/D1+	136	64.5
D2	75	35.5
Node dissection according to guidelines		
Standard	183	86.7
Reduced	28	13.3
Operative duration (min)	319.3 ± 79.7 (140‐573)	
Operative blood loss (mL)	275.4 ± 346.1 (0‐2040)	
pStage		
I	134	63.5
II	39	18.5
III	38	18.0
Histology		
Differentiated	151	71.6
Undifferentiated	60	28.4
Adjuvant chemotherapy		
Negative	172	81.5
Positive	39	18.5
Postoperative complication		
Negative	151	71.6
Positive	60	28.4
Postoperative infectious complication		
Negative	173	82.0
Positive	38	18.0
Hospital stay (days)	22.3 ± 17.6 (9‐120)	

*Note:* Data are presented as mean ± SD (range) or number.

Abbreviations: BMI, body mass index; NLR, neutrophil‐to‐lymphocyte ratio; PLR, platelet‐to‐lymphocyte; PNI, prognostic nutritional index; PS, performance status; SMI, skeletal muscle index ratio; VFA, visceral fat area.

### 3.3 Survival outcome

The overall 5‐year survival rate was 61.4%, the NGCaS was 70.2% for the entire cohort, and the median follow‐up period for survivors was 62 months. At the time of the analysis, 21 patients (10.0%) had died of gastric cancer and 60 patients (28.4%) had died of non‐gastric‐cancer‐related causes. Table 2 shows the causes of death within 5 years for each stage. Overall, within 5 years, 21 deaths were from gastric cancer and 50 deaths were non‐gastric cancer related. Details of the non‐gastric‐cancer‐related deaths were known in 54 patients: pneumonia in 18 patients, cardiovascular disease in 11 patients, other carcinoma in eight patients, cerebrovascular disease in three patients, liver disease in three patients, senility in three patients, suicide in two patients, gastrointestinal hemorrhage in one patient, renal failure in one patient, sepsis in one patient, bedsore in one patient, trauma in one patient, and asphyxia in one patient. Tumor recurrence occurred in 25 patients.

**TABLE 2:** Cause of death within 5 years after gastrectomy

Cause of death	
pStage I	(n = 134)
Gastric cancer	2 (1.5%)
Other disease	25 (18.6%)
pStage II	(n = 39)
Gastric cancer	5 (12.8%)
Other disease	16 (41.0%)
pStage III	(n = 38)
Gastric cancer	14 (36.8%)
Other disease	9 (23.6%)

### 3.4 Prognostic factors in perioperative parameters for OS

Results of the univariate analyses for OS are summarized in Tables 3. Univariate analyses revealed that among the perioperative factors, sex, mFI, CCI, PNI, NLR, SMI, preoperative T factor, extent of resection, surgical approach, node dissection according to guidelines, blood loss, pathological T factor, pathological N factor, TMN stage, and presence or absence of adjuvant chemotherapy were significantly associated with OS. Multivariate analysis was performed for preoperative factors only and all perioperative factors, respectively, and are summarized in Table 4. Multivariate analyses of preoperative parameters with *P* < 0.05 in the univariate analyses revealed that sex, NLR, and SMI were independent risk factors for OS (HR 2.338, 95% CI 1.322‐4.134, *P* = 0.003, HR 2.241, 95% CI, 1.352‐3.715, *P* = 0.002, HR 2.119, 95% CI, 1.277‐3.516, *P* = 0.004, respectively). Multivariate analyses of all perioperative parameters with *P* < 0.05 in the univariate analyses revealed that sex and NLR were independent risk factors for OS (HR 2.386, 95% CI 1.329‐4.281, *P* = 0.004, HR 1.938, 95% CI 1.133‐3.314, *P* = 0.016).

**TABLE 3:** Univariate analyses of prognostic factors for OS of elderly patients

Variables	No. of patients	5‐year OS(%)	*P* value
	n		
Preoperative factor			
Age			0.059
≤79	114	67.7	
>79	97	52.7	
Sex			0.004*
Male	136	53.7	
Female	75	74.6	
PS			0.407
0	159	61.1	
≥1	52	62.1	
Modified frailty index			0.032*
≤1	127	68.2	
>1	84	52.1	
Charlson comorbidity index			0.007*
≤1	141	68.1	
>1	70	47	
PNI			0.000*
≤45.06	77	42	
>45.06	134	72.2	
NLR			0.000*
≤2.24	106	76.2	
>2.24	105	46.7	
PLR			0.011*
≤145.99	123	68.7	
>145.99	88	51	
BMI			0.080
≤21.53	81	54.6	
>21.53	130	66.8	
VFA			0.428
≤111.5	100	57.5	
>111.5	111	63.8	
SMI			0.012*
≤38.49	74	48.5	
>38.49	137	67.4	
Preoperative T factor			0.000*
T1	130	72.9	
>T2	81	44.7	
Preoperative N factor			0.033*
N0	169	65.4	
≥N1	42	46.1	
Operation, pathology, and postoperative factor			
Extent of resection			0.003*
Distal/Proximal gastrectomy	148	68.3	
Total gastrectomy	63	44.3	
Approach			0.000*
Open	68	36.2	
Laparoscopy	143	75.3	
Lymph node dissection			0.403
D1/D1+	136	64.9	
D2	75	55.6	
Node dissection according to guidelines			0.000*
Standard	183	66.2	
Reduced	28	35.9	
Operative duration			0.899
≤296 min	88	60.4	
>296 min	123	62	
Blood loss			0.004*
≤140 mL	105	70.9	
>140 mL	106	52.3	
Histology			0.461
Differentiated	151	63.8	
Undifferentiated	60	55.5	
Pathological T factor			0.000*
T1	134	74.7	
>T2	77	39.6	
Pathological N factor			0.001*
N0	140	69.3	
≥N1	71	45.7	
TMN stage			0.000*
I	134	74.7	
II, III	77	39.6	
Adjuvant chemotherapy			0.043*
Negative	172	65.4	
Positive	39	45.3	
Postoperative complication			0.318
Negative	151	63.5	
Positive	60	56	
Postoperative infectious complication			0.162
Negative	173	63.6	
Positive	38	49.1	

Abbreviations: BMI, body mass index; NLR, neutrophil‐to‐lymphocyte ratio; OS, Overall survival; PLR, platelet‐to‐lymphocyte ratio; PNI, prognostic nutritional index; PS, performance status; SMI, skeletal muscle index; VFA, visceral fat area.

* Statistical significance (*P* < 0.05).

**TABLE 4:** Multivariate analyses for OS of elderly patients

Variables	All perioperative factors		Preoperative factors limited	
	HR (95% CI)	*P* value	HR (95% CI)	*P* value
Sex		0.004*		0.003*
Male	2.386 (1.329‐4.281)		2.338 (1.322‐4.134)	
Female	1		1	
Modified frailty index		0.482		0.2338
≤1	1		1	
>1	1.195 (0.727‐1.965)		1.326 (0.829‐2.118)	
Charlson comorbidity index		0.144		0.071
≤1	1		1	
>1	1.483 (0.875‐2.515)		1.595 (0.961‐2.645)	
PNI		0.723		0.313
≤45.06	1.116 (0.608‐2.048)		1.328 (0.765‐2.304)	
>45.06	1		1	
NLR		0.016*		0.002*
≤2.24	1		1	
>2.24	1.938 (1.133‐3.314)		2.241 (1.352‐‐3.715)	
PLR		0.979		0.940
≤145.99	1.007 (0.580‐1.749)		1.020 (0.603‐1.726)	
>145.99	1		1	
SMI		0.094		0.004*
≤38.49	1.638 (0.919‐2.918)		2.119 (1.277‐3.516)	
>38.49	1		1	
Preoperative T factor		0.818		0.066
T1	1		1	
>T2	1.107 (0.467‐2.621)		1.706 (0.965‐3.017)	
Preoperative N factor		0.776		0.965
N0	1		1	
≥N1	1.107 (0.548‐2.236)		1.041 (0.568‐1.910)	
Extent of resection		0.235		
Distal/Proximal gastrectomy	1			
Total gastrectomy	1.345 (0.825‐2.192)			
Approach		0.152		
Open	1.786 (0.808‐3.945)			
Laparoscopy	1			
Node dissection		0.245		
Standard	1			
Reduced	1.512 (0.753‐3.034)			
Blood loss		0.482		
≤140 mL	1			
>140 mL	1.272 (0.650‐2.490)			
Pathological T factor		0.328		
T1	1			
>T2	1.700 (0.588‐4.918)			
Pathological N factor		0.322		
N0	1			
≥N1	1.455 (0.692‐3.056)			
TMN stage		0.174		
I	1			
II,III	2.182 (0.708‐6.726)			
Adjuvant chemotherapy		0.315		
Negative	1			
Positive	1.464 (0.696‐3.076)			

*Note:* Node dissection lymph node dissection according to guidelines.

Abbreviations: NLR, neutrophil‐to‐lymphocyte ratio; OS, overall survival; PLR, platelet‐to‐lymphocyte ratio; PNI, prognostic nutritional index; SMI, skeletal muscle index.

* Statistical significance (*P* < 0.05).

### 3.5 Prognostic factors in perioperative parameters for NGCaS

Results of univariate analyses for NGCaS are summarized in Table 5. Univariate analyses revealed that among perioperative factors, sex, mFI, CCI, PNI, NLR, SMI, surgical approach, node dissection according to guidelines, and TMN stage were significantly associated with NGCaS. Multivariate analysis was performed for preoperative factors only and all perioperative factors, respectively, and are summarized in Table 6. Multivariate analyses of only preoperative parameters with *P* < 0.05 in the univariate analyses revealed that sex, NLR, and SMI were independent risk factors for NGCaS (HR 2.493, 95% CI, 1.297‐4.793, *P* = 0.006, HR 1.958, 95% CI, 1.131‐3.388, *P* = 0.016, HR 2.594, 95% CI, 1.467‐4.589, *P* = 0.001). Multivariate analyses of all perioperative parameters with *P* < 0.05 in the univariate analyses revealed that sex, NLR, SMI, and node dissection according to guidelines were independent risk factors for NGCaS (HR 2.303, 95% CI 1.1974.429, *P* = 0.012, HR 1.814, 95% CI 1.024‐3.212, *P* = 0.041, HR 1.947, 95% CI 1.029‐3.685, *P* = 0.041, HR 2.036, 95% CI 1.021‐4.060, *P* = 0.043).

**TABLE 5:** Univariate analyses of prognostic factors for NGCaS of elderly patients

Variables	No. of patients	5‐year NGCaS (%)	*P* value
	n		
Preoperative factor			
Age			0.053
≤79	114	75.5	
>79	97	63.1	
Sex			0.010*
Male	136	63.7	
Female	75	80.9	
PS			0.076
0	159	71.4	
≥1	52	65.6	
Modified frailty index			0.023*
≤1	127	77	
>1	84	60.7	
Charlson comorbidity index			0.010*
≤1	141	76.6	
>1	70	55.8	
PNI			0.004*
≤45.06	77	53.9	
>45.06	134	78.2	
NLR			0.003*
≤2.25	106	80.5	
>2.25	105	58.2	
PLR			0.099
≤146.88	123	75.4	
>146.88	88	62	
BMI			0.32
≤20.96	81	64.7	
>20.96	130	72.1	
VFA			0.114
≤115.5	100	65.1	
>115.5	111	74.2	
SMI			0.023*
≤38.49	74	57.9	
>38.49	137	75.7	
Preoperative T factor			0.072
T1	130	76.1	
>T2	81	60.4	
Preoperative N factor			0.607
N0	169	69.8	
≥N1	42	72.4	
Operation, pathology, and postoperative factor			
Extent of resection			0.078
Distal/Proximal gastrectomy	148	73.6	
Total gastrectomy	63	61.5	
Approach			0.003*
Open	68	51.6	
Laparoscopy	143	78.8	
Lymph node dissection			0.335
D1/D1+	136	69.5	
D2	75	71.4	
Node dissection according to guidelines			0.000*
Standard	183	75.1	
Reduced	28	43.7	
Operative duration			0.726
≤296 min	88	68.8	
>296 min	123	71.1	
Blood loss			0.119
≤140 mL	105	74.8	
>140 mL	106	65.1	
Histology			0.514
Differentiated	151	69.5	
Undifferentiated	60	72.1	
Pathological T factor			0.063
T1	132	75.6	
>T2	79	60.1	
Pathological N factor			0.689
N0	140	71	
≥N1	71	68.6	
TMN stage			0.037*
I	134	76.6	
II, III	77	57.3	
Adjuvant chemotherapy			0.466
Negative	172	69.9	
Positive	39	71.2	
Postoperative complication			0.318
Negative	151	73	
Positive	60	62.7	
Postoperative infectious complication			0.162
Negative	173	72.4	
Positive	38	57.2	

Abbreviations: NGCaS, Non‐gastric‐cancer‐related survival; PS, performance status; PNI, prognostic nutritional index; NLR, neutrophil‐to‐lymphocyte ratio; PLR, platelet‐to‐lymphocyte ratio; BMI, body mass index; VFA, visceral fat area, SMI, skeletal muscle index.

* Statistical significance (*P* < 0.05).

**TABLE 6:** Multivariate analyses for NGCaS by perioperative factors and preoperative factors

Variables	All perioperative factors		Preoperative factors limited	
	HR (95% CI)	*P* value	HR (95% CI)	*P* value
Sex		0.012*		0.006*
Male	2.303 (1.197‐4.429)		2.493 (1.297‐4.793)	
Female	1		1	
Modified frailty index		0.127		0.061
≤1	1		1	
>1	1.563 (0.881‐2.773)		1.671 (0.997‐2859)	
Charlson comorbidity index		0.195		0.100
≤1	1		1	
>1	1.485 (0.817‐2.699)		1.628 (0.911‐2.910)	
PNI		0.766		0.352
≤45.06	1.101 (0.584‐2.075)		1.303 (0.746‐2.276)	
>45.06	1		1	
NLR		0.041*		0.016*
≤2.25	1		1	
>2.25	1.814 (1.024‐3.212)		1.958 (1.131‐3.388)	
SMI		0.041*		0.001*
≤38.49	1.947 (1.029‐3.685)		2.594 (1.467‐4.589)	
>38.49	1		1	
Approach		0.628		
Open	1.212 (0.556‐2.642)			
Laparoscopy	1			
Node dissection		0.043*		
Standard	1			
Reduced	2.036 (1.021‐4.060)			
TMN stage		0.800		
I	1			
II, III	1.100 (0.525‐2.306)			

*Note:* Node dissection lymph node dissection according to guidelines.

Abbreviations: 95% CI, 95% confidence interval; HR, hazard ratio; NGCaS, Non‐gastric‐cancer‐related survival; NLR, neutrophil‐to‐lymphocyte ratio; PNI, prognostic nutritional index; SMI, skeletal muscle index.

* Statistical significance (*P* < 0.05).

**FIGURE 1:**
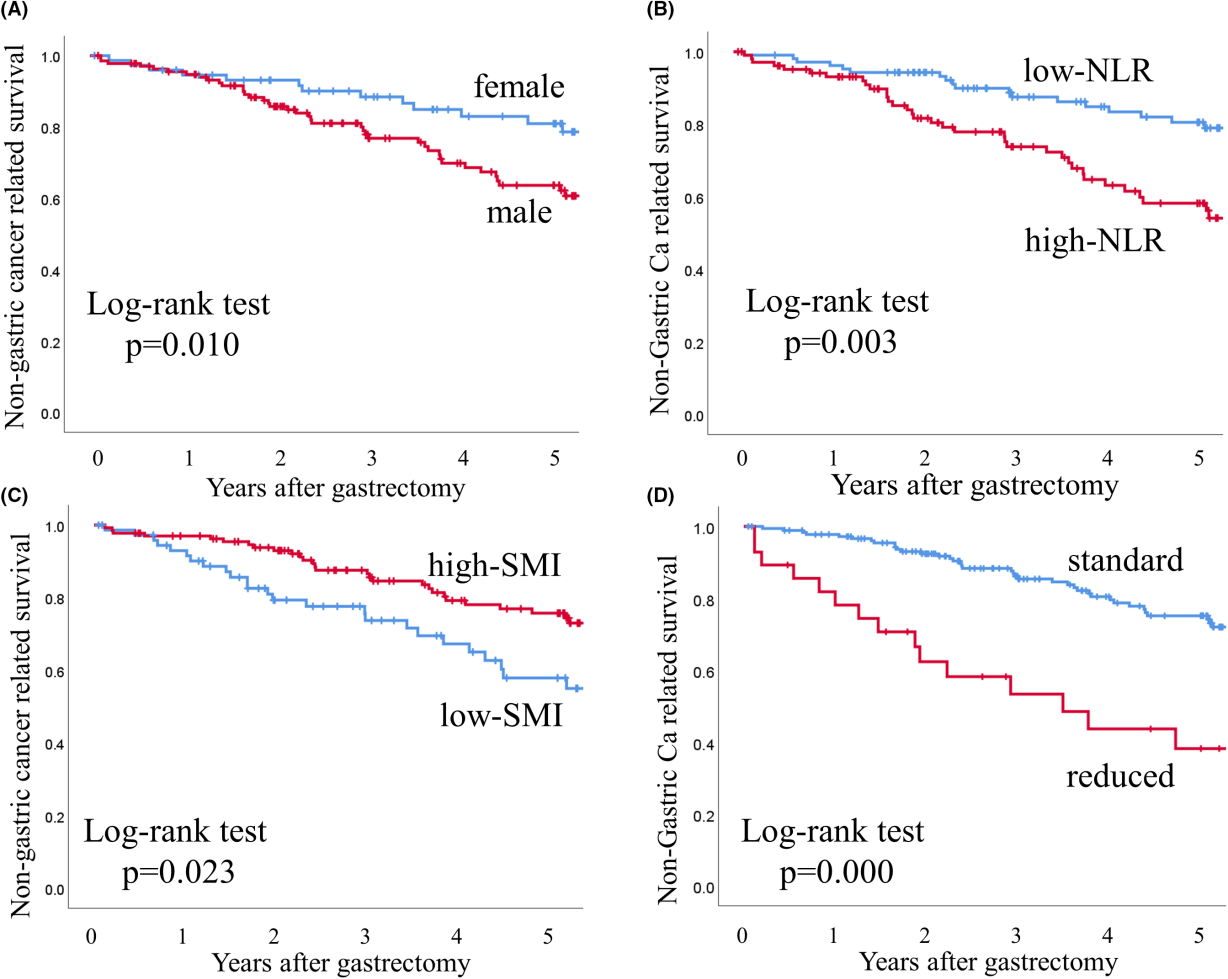
Kaplan‐Meier survival curves for non‐gastric‐cancer‐related survival (NGCaS) according to each independent risk factors. (A) The 5‐year NGCaS rates in the groups for men and women were 63.7% and 80.9%, respectively (*P* = 0.010). (B) The five‐year NGCaS rates in the groups for patients with low and high NLR were 80.5% and 58.2%, respectively (*P* = 0.003). (C) The 5‐year NGCaS rates in the groups for patients with low and high SMI were 57.9% and 75.7%, respectively (*P* = 0.023). (D) The five‐year NGCaS rates in the groups for patients with standard and reduced node dissection according to guidelines were 75.1% and 43.7%, respectively (*P* = 0.000)

Figure 1 shows Kaplan‐Meier survival curves for NGCaS according to each independent risk factor. The 5‐year OS rates in the groups were 62.7% and 80.7% for males and females (*P* = 0.010), respectively, 81.1% and 57.0% for those with low and high NLR (*P* = 0.003), respectively, 57.2% and 75.5% for those with low and high SMI (*P* = 0.023), respectively, and 75.1% and 43.7% for those with standard and reduced lymph node dissection according to guidelines (*P* = 0.000), respectively.

### 3.6 NGCaS by number of positive risk factors

**FIGURE 2:**
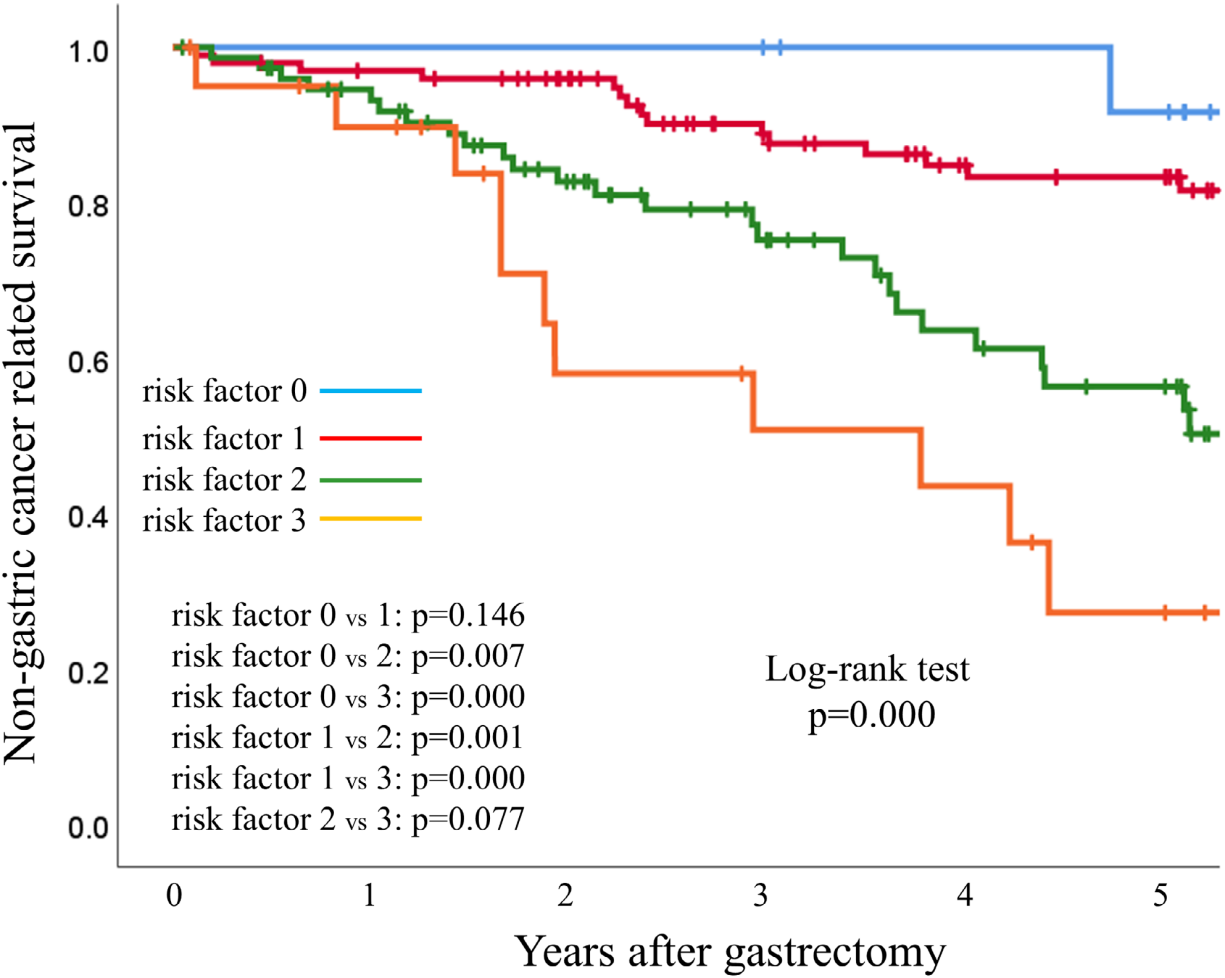
Kaplan–Meier survival curves for non‐gastric‐cancer‐related survival (NGCaS) according to number of positive risk factors. The 5‐year NGCaS of each group were 91.7% (risk factor 0), 83.3% (risk factor 1), 56.3% (risk factor 2), and 27.2% (risk factor 3) (*P* < 0.001)

We stratified the NGCaS using number of positive preoperative factors detected by the multivariate analysis (sex = positive for man; NLR = positive for >2.25; SMI = positive for ≤38.5). Patients were divided into four categories according to the number of risk factors as follows: risk factor 0 (none positive risk factors), risk factor 1 (one positive risk factor), risk factor 2 (two positive risk factors), risk factor 3 (three positive risk factors). The 5‐year NGSS in risk factor 0 (N = 14), risk factor 2 (N = 100), risk factor 3(N = 76), risk factor 4 (N = 21) group were 91.7%, 83.3%, 56.3% and 27.2%, respectively (*P* < 0.001) (Figure. 2).

## 4 DISCUSSION

In this study, we attempted to identify patients at a high risk for death, especially non‐gastric‐cancer‐related death, after gastrectomy for gastric cancer in patients aged >75 years. The results showed that the risk of non‐gastric‐cancer‐related death increased with preoperative risk factors of male sex, low SMI, and high NLR. These results may be useful in the development of treatment strategies for elderly patients with gastric cancer, considering not only the risk of death from gastric cancer but also the risk of death from other diseases.

Until recently, reports analyzing data on the survival of cancer patients have focused on cancer‐related death, with little consideration given to non‐cancer‐related death.^17^ However, since the rate of non‐cancer‐related death is higher in elderly patients compared to young patients, the prediction of postoperative non‐cancer‐related death may be important for some types of cancer surgeries in elderly patients. According to the national registry of the Japanese gastric cancer association (JGCA), the 5‐year OS was 47.0%‐93.1% and disease‐specific survival (DSS) was 91.4%‐98.2% after gastrectomy for stage I gastric cancer in patients aged >75 years. The ratio of non‐gastric‐cancer‐related death to total deaths was very high.^9^ In Japan, it has been reported that about 70% of cases of radical gastrectomy for gastric cancer were for stage I of gastric cancer.^18^ In short, because a high proportion of gastric cancer surgery patients in Japan are stage I cancer cases, the proportion of non‐gastric‐cancer‐related death in elderly patients with gastric cancer is relatively high. In the report by Hashimoto et al, among the gastric cancer patients who underwent radical gastrectomy at >75 years of age, 64.3% were stage I cancer patients, and 73.9% of deaths within 5 years after surgery were due to non‐gastric‐cancer‐related reasons.^12^ In this study, 63.5% of the cases were stage I cancer cases, and 70.4% of deaths within 5 years after surgery were due to non‐gastric‐cancer‐related reasons, similar to the aforementioned report. Furthermore, the identified three risk factors for NGCaS were also identified as risk factors for the OS in the multivariate analysis, suggesting a strong impact of NGCaS in the elderly.

In our study, among the preoperative factors for gastric cancer in the elderly, male sex, low SMI, and high NLR were shown to be independent risk factors for NGCaS after gastrectomy. With regard to the sex of the patients, Japanese government data shows that the life expectancy at 75 years of age is 12.6 years for men and 16.3 years for women, with men having a shorter life expectancy regardless of whether they have gastric cancer.^19^ JGCA data also showed that in gastric cancer patients aged >75 years, non‐gastric‐cancer‐related deaths were more common in men than in women in each age group,^9^ so it is reasonable that men were selected as a risk factor for the NGCaS.

Sarcopenia has been reported to correlate closely to functional impairment, physical disability, and even increased risk of death.^20^ SMI is an objective index of sarcopenia calculated using CT and has been reported to be associated with the prognosis in solid tumors,^21^ and has also been reported to be associated with the postoperative survival in gastric cancer.^22^, ^23^ Kuwada et al reported that non‐gastric‐cancer‐related death after gastric cancer surgery is more common in sarcopenic patients with comorbidities,^13^ and that SMI, which can be easily measured by CT, may also be a risk factor for non‐gastric‐cancer‐related death after gastric cancer surgery in the elderly. The NLR is one of the inflammation‐related parameters along with the PNI and PLR, and has been reported as a prognostic factor among various cancer patients.^24^, ^25^, ^26^, ^27^ Although there were no reports showing an association between the NLR and non‐gastric‐cancer‐related death, the PNI, one of the inflammatory markers, was shown to be associated with death from other diseases after gastric cancer surgery.^12^, ^28^ In the present study, the PNI and NLR, both inflammation‐based markers, were associated with NGCaS in the univariate analysis. However, in the multivariate analysis, only the NLR was identified as an independent risk factor for death from other diseases, not the PNI. Although the mechanism of the association between inflammation‐based markers and death from other diseases is unclear, our results suggest that the NLR, an inflammatory marker, may also be a prognostic factor for death from other diseases.

Among intraoperative and postoperative factors, non‐guideline‐compliant lymph node dissection was identified as a risk factor for NGCaS. However, the extent of lymph node dissection did not affect NGCaS, suggesting that reduced lymph node dissection did not affect NGCaS, but rather that reduced lymph node dissection was performed in patients with poor prognosis.

In this study, while male sex, sarcopenia, and NLR were identified as risk factors for NGCaS, age was not identified as a significant risk factor for NGCaS. Although it has been reported that non‐gastric‐cancer‐related death after gastrectomy increases with age,^9^ there is currently no fixed cutoff for age, as various values have been reported.^29^, ^30^, ^31^ This indicates that it is difficult to predict the postoperative life expectancy of postoperative gastric cancer patients based on age alone, and to formulate treatment strategies based on that.

This study also showed that patients with multiple risk factors had a higher mortality rate from death due to other diseases. This indicates that combining multiple risk factors may better predict death from other diseases. Generally, in survival prediction based on multiple risk factors, risk factors are often weighted by hazard ratio and the total score obtained by adding the hazard ratios of positive risk factors may be used.^32^ Since the hazard ratios of the three risk factors in this study were in the close range of 1.96‐2.59, we simplified them by using the number of risk factors rather than the total score obtained by adding the hazard ratios of the three risk factors. However, more precise scoring is an issue to be addressed in the future.

There are several potential limitations of this study. First, this was a retrospective study and the number of patients was relatively small. Individual risk factors for NGCaS and NGCaS by number of positive risk factors have to be validated in prospective studies with a large number of patients. Second, the analysis of risk factors for NGCaS after gastrectomy used factors from preoperative examinations usually performed before gastrectomy, such as past history, blood tests, and imaging studies, but did not include items from the detailed functional assessment of elderly patients, such as a comprehensive geriatric assessment.^33^, ^34^, ^35^ Third, in this study, we cannot determine whether the patients who were found to be at high risk for non‐gastric‐cancer‐related death were originally a group of patients with a short life expectancy or whether the surgery had a negative impact on their prognosis. To clarify these points, a background‐matched study comparing the prognosis of elderly patients who underwent gastrectomy with those who did not undergo surgery in a large number of patients is needed.

In conclusion, among the common preoperative factors obtained before gastrectomy, male sex, low SMI, and high NLR are risk factors for non‐gastric‐cancer‐related death after gastrectomy. Patients with more than one of these factors are at a higher risk for non‐gastric‐cancer‐related death after gastrectomy and require a careful treatment strategy and mid‐ to long‐term postoperative follow‐up.

## DISCLOSURE

Funding: The authors received no specific funding for this study.

Conflict of Interest: Hiroaki Nagano is an editorial board member of *Annals of Gastroenterological Surgery*.

Ethical Considerations: This study was approved by the institutional review board of the Yamaguchi University Hospital (H28‐182). All informed consent was obtained from the patients. Registry and the Registration No. of the study: N/A Trial. Animal Studies: N/A.

Author Contributions: Michihisa Iida and Shigeru Takeda conceived of the presented idea. Michihisa Iida wrote the manuscript. Chiyo Nakashima, Mitsuo Nishiyama, Yusaku Watanabe, and Shigefumi Yoshino contributed to data collection. Yuki Nakagami and Tsuyoshi Tanabe performed the analytic calculations. Nobuaki Suzuki aided in interpreting the results. Hiroaki Nagano supervised the project. All authors provided critical feedback and helped shape the research, analysis, and manuscript.

## Supporting information


Figure S1

Figure S2
