# Supplementary figures and images for: Risk factors for non‐gastric‐cancer‐related death after gastrectomy in elderly patients
Source: Ann Gastroenterol Surg. 2022 Jun 20;6(6):753–66. doi: 10.1002/ags3.12588 (PMC9628222; doi:10.1002/ags3.12588)

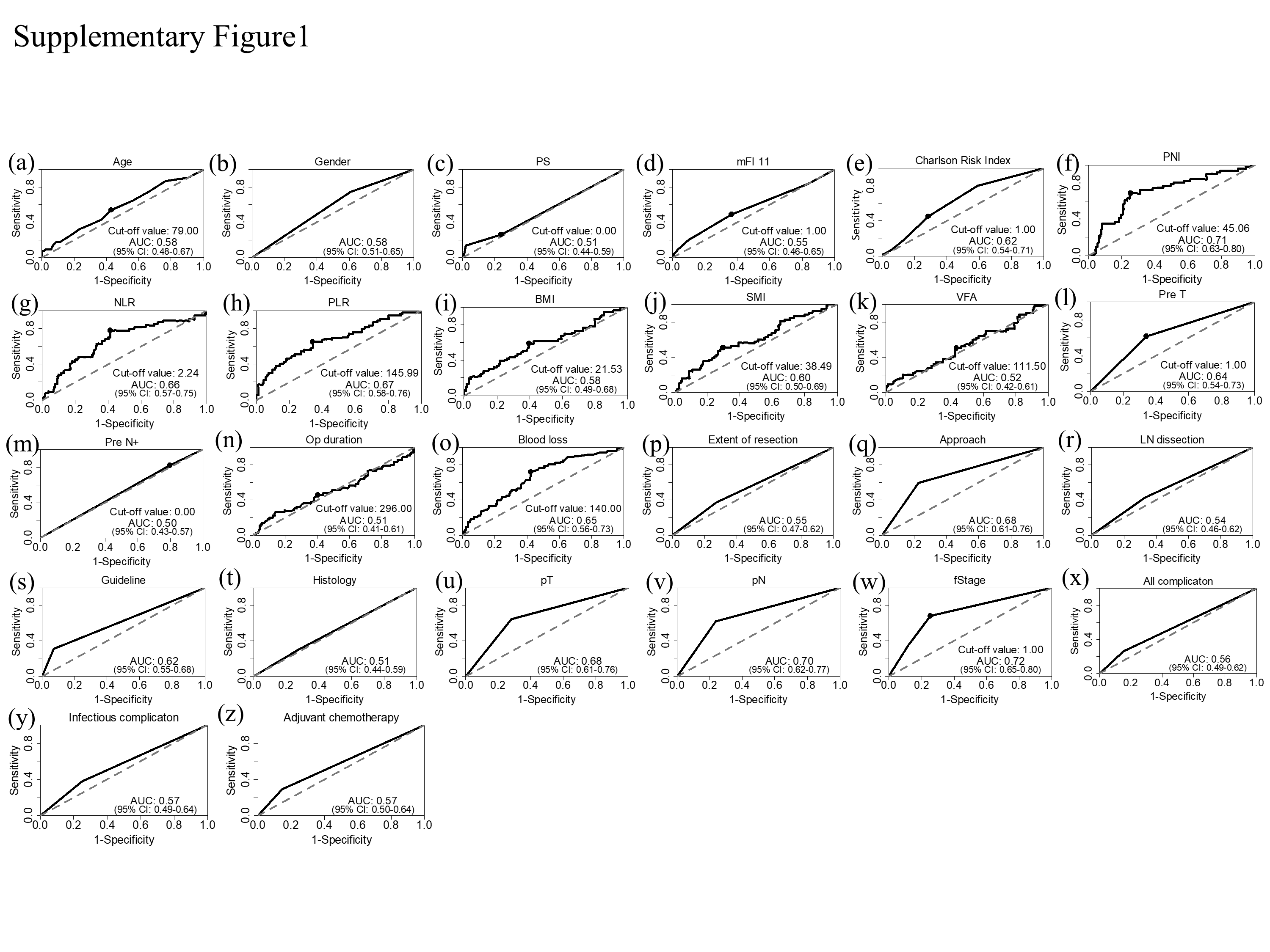

Supplement: Supplementary file 1 — Figure S1 [file AGS3-6-753-s002.TIF]

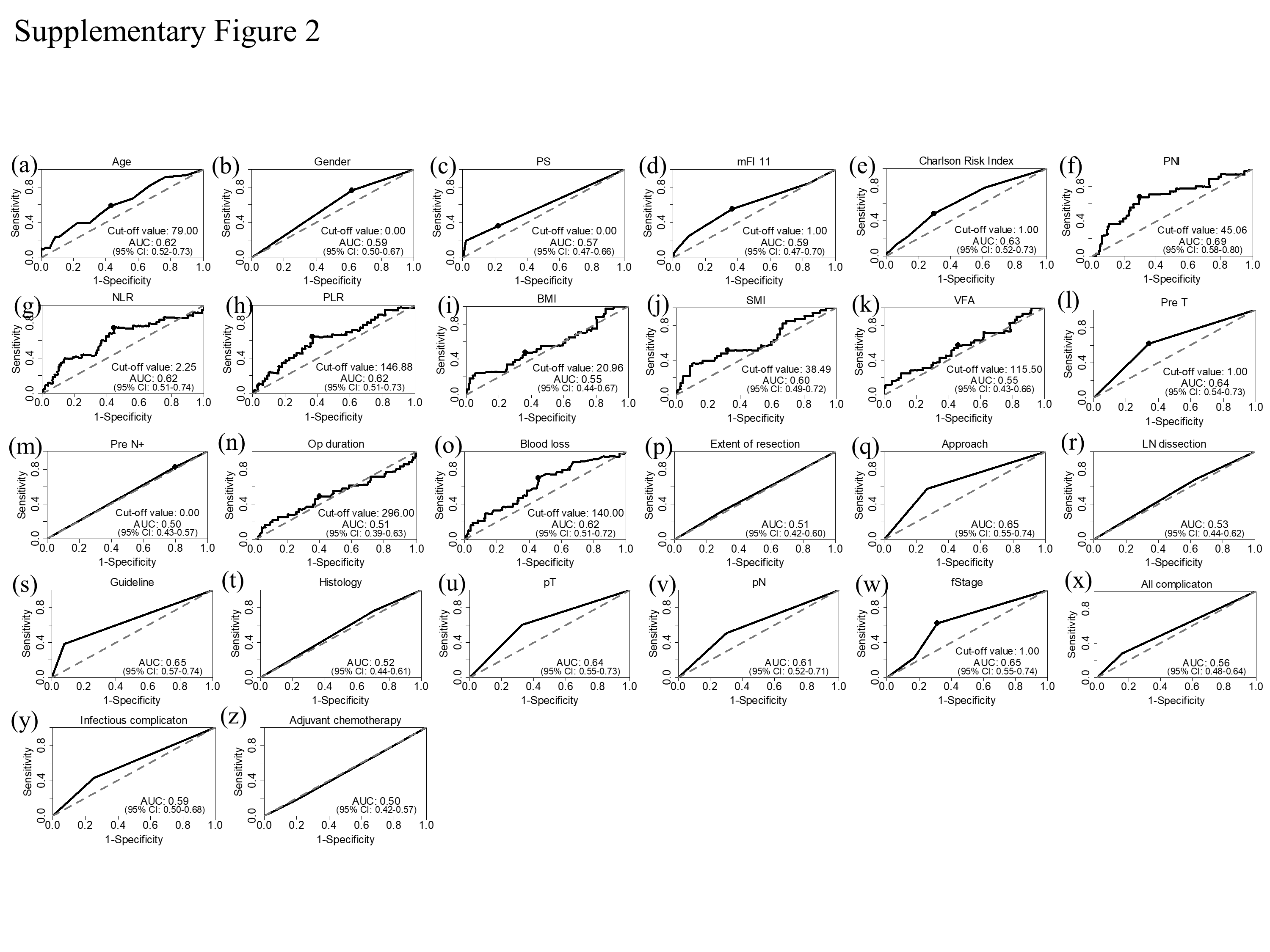

Supplement: Supplementary file 2 — Figure S2 [file AGS3-6-753-s001.TIF]
